# Huangjin Shuangshen decoction alleviates chronic atrophic gastritis by suppressing TNF/NF-κB signaling and promoting CFTR-associated gastric mucosal barrier repair

**DOI:** 10.3389/fimmu.2026.1848471

**Published:** 2026-07-10

**Authors:** Shuo Zhang, Shuya Zhang, Mengyi Wang, Xueru Huang, Chen Huang, Tao Jiang, Guangji Zhang

**Affiliations:** 1 School of Basic Medical Sciences, Zhejiang Chinese Medical University, Hangzhou, China; 2 Zhejiang Key Laboratory of Blood-Stasis-Toxin Syndrome, Zhejiang Chinese Medical University, Hangzhou, China; 3 Traditional Chinese Medicine “Preventing Disease” Wisdom Health Project Research Center of Zhejiang, Hangzhou, China

**Keywords:** CFTR, chronic atrophic gastritis, gastric mucosal barrier repair, Huangjin Shuangshen decoction, inflammation–barrier crosstalk, TNF/NF-κB signaling

## Abstract

**Background:**

Chronic atrophic gastritis (CAG) is a precancerous gastric lesion characterized by persistent inflammatory injury, glandular atrophy, and impairment of gastric mucosal barrier-associated integrity. Huangjin Shuangshen Decoction (HJSS) has shown therapeutic potential in gastritis-related disorders, but its effects on CAG and the underlying mechanism remain unclear. This study investigated whether HJSS alleviates CAG by modulating inflammation-associated barrier dysfunction.

**Methods:**

CAG was induced in mice by MNNG combined with ranitidine and irregular feeding, followed by treatment with different doses of HJSS, with folic acid as a positive control. Histopathology, gastric function indices, inflammatory mediators, apoptosis-related markers, and barrier-associated molecules were assessed *in vivo*. MNNG-injured GES-1 cells treated with HJSS-medicated serum were used for *in vitro* validation. Transcriptomic analysis, network pharmacology, and pharmacological inhibition were integrated to explore the underlying mechanisms.

**Results:**

HJSS alleviated gastric mucosal atrophy and histopathological injury, improved gastric functional impairment, reduced inflammatory burden, and attenuated apoptosis-associated epithelial injury in experimental CAG. HJSS also promoted the recovery of gastric mucosal barrier-associated molecules, including CFTR, ZO-1, MUC5AC, Occludin, and Claudin-1. Integrated transcriptomic and network pharmacology analyses highlighted an inflammation–barrier framework involving TNF-related signaling and CFTR-associated regulation. *In vitro*, HJSS mitigated MNNG-induced epithelial injury, whereas CFTR inhibition attenuated the HJSS-associated restoration of CFTR and ZO-1. HJSS was further associated with suppression of TNF/NF-κB signaling and reduced p65 nuclear translocation.

**Conclusion:**

HJSS alleviates MNNG-induced CAG by attenuating inflammatory injury and promoting the recovery of gastric mucosal barrier-associated molecular features. Its protective effects are associated with suppression of TNF/NF-κB signaling and involvement of CFTR-associated regulation, supporting an inflammation–barrier mechanism underlying the action of HJSS in CAG.

## 1 Introduction

Chronic atrophic gastritis (CAG) is a well-recognized precancerous gastric lesion within the Correa cascade and remains a major clinical concern because of its progressive evolution toward intestinal metaplasia, dysplasia, and gastric cancer (1, 2). Histopathologically, CAG is characterized by chronic inflammatory infiltration, progressive gland loss, mucosal thinning, and impairment of gastric secretory function (3–5). Although current management strategies, including acid suppressants, mucosal protectants, Helicobacter pylori eradication, and folate supplementation, may provide partial benefit, effective interventions for preventing or delaying CAG progression remain limited (6).

Increasing evidence suggests that CAG should not be viewed solely as a chronic inflammatory disorder, but also as a disease involving impaired gastric mucosal barrier-associated integrity. The integrity of the gastric mucosa depends on coordinated maintenance of the mucus layer, epithelial junctional complexes, and epithelial homeostasis-associated molecules (7–9). Once this barrier is compromised, luminal irritants and inflammatory stimuli can further aggravate mucosal injury, thereby generating a self-perpetuating cycle between persistent inflammatory activation and epithelial barrier disruption (10, 11). In this context, therapeutic strategies capable of simultaneously attenuating inflammatory injury and supporting mucosal barrier-associated repair may be particularly relevant to disease control.

Among the signaling pathways implicated in chronic gastric injury, TNF/NF-κB signaling is a central regulator of inflammatory amplification, epithelial stress, and mucosal damage. Sustained activation of NF-κB promotes the expression of pro-inflammatory mediators and contributes to epithelial injury in chronic gastric disorders (12, 13). In parallel, cystic fibrosis transmembrane conductance regulator (CFTR), beyond its classical role as an ion channel, has increasingly been implicated in epithelial homeostasis and barrier regulation (14). CFTR has been shown to interact with ZO-1 and to participate in tight-junction assembly and epithelial differentiation through the ZO-1–ZONAB pathway, suggesting that it may represent a functionally relevant node linking ion transport, junctional organization, and epithelial integrity (15). Barrier-associated molecules such as Occludin, Claudin-1, and MUC5AC are likewise essential for maintaining gastric epithelial organization (16). However, whether CFTR-associated regulation contributes to inflammation-related barrier alterations in CAG remains insufficiently understood.

Multi-component herbal formulas may offer potential advantages in chronic gastric disorders because they can simultaneously modulate several pathological processes, including inflammatory signaling and epithelial injury (17, 18). Nevertheless, although accumulating studies suggest that traditional Chinese medicine (TCM) formulas can improve CAG phenotypes through inflammation-related pathways, mechanistic validation of the molecular nodes connecting inflammatory suppression to barrier repair is often insufficient (19, 20). Huangjin Shuangshen Decoction (HJSS), a team-developed modified formula derived from the classical formula Si-Miao-Yong-An Decoction with the addition of *Astragalus membranaceus*, *Salvia miltiorrhiza*, and *Actinidia chinensis*, has been applied in the gastritis–carcinogenesis continuum (21, 22). From a TCM perspective, chronic gastritis progression is considered to involve spleen–stomach deficiency as the underlying basis and blood stasis–toxin accumulation as a key pathological feature. Accordingly, HJSS was developed under the therapeutic principles of tonifying Qi and strengthening the spleen, activating blood circulation and resolving stasis, clearing heat and toxins, and protecting the gastric mucosa. In this formula, *Astragalus membranaceus* reinforces Qi, *Salvia miltiorrhiza* and *Angelica sinensis* promote blood circulation and nourish blood, *Lonicera japonica*, *Actinidia chinensis*, and *Scrophularia ningpoensis* clear heat, remove toxins, and soften stagnation, while *Glycyrrhiza uralensis* harmonizes the formula. However, its effects on chronic atrophic gastritis, particularly from the perspective of coordinated inflammatory control and barrier preservation, remain insufficiently defined.

In the present study, we investigated the protective effects of HJSS in an MNNG-induced CAG model *in vivo* and in MNNG-injured gastric epithelial cells *in vitro*. By integrating histopathological evaluation, functional assessment, transcriptomic profiling, network pharmacology, and pharmacological validation, we aimed to determine whether HJSS alleviates CAG by attenuating inflammatory injury and promoting the recovery of gastric mucosal barrier-associated molecular integrity, with particular attention to TNF/NF-κB signaling and CFTR-associated regulation.

## 2 Methods

### 2.1 Preparation of HJSS and LC–MS fingerprinting

HJSS consists of seven herbal medicines; the botanical names and dosages are provided in **Table 1**. HJSS was prepared by standardized water extraction, filtration, and concentration for intragastric administration. Low-, medium-, and high-dose HJSS groups were defined according to body surface area conversion. For quality control, HJSS was characterized by LC–MS under both positive and negative electrospray ionization modes to generate total ion chromatograms (TICs), providing global chemical profiling and batch consistency assessment.

**Table 1:** Contents of HJSS decoction.

TCM name	Latin name	Part used	Amount (g)
Huangqi	Astragalus membranaceus (Fisch.) Bunge	Root	30
Jinyinhua	Lonicera japonica Thunb.	Flower	15
Xuanshen	Scrophularia ningpoensis Hemsl.	Root	12
Danshen	Salvia miltiorrhiza Bunge	Root and rhizome	15
Danggui	Angelica sinensis (Oliv.) Diels	Root	10
Tengligen	Actinidia chinensis Planch.	Root and rhizome	15
Gancao	Glycyrrhiza uralensis Fisch. ex DC.	Root and rhizome	6

### 2.2 Animals, model establishment, and treatment

Male KM mice (4 weeks old, 20–30 g) were obtained from Hangzhou Qizhen Laboratory Animal Technology Co., Ltd. (Hangzhou, China) and housed under specific pathogen-free conditions (22 ± 1 °C, 50–70% humidity, 12-h light/dark cycle) with ad libitum access to standard chow and water. All animal procedures were approved by the Institutional Animal Care and Use Committee of Zhejiang Chinese Medical University (IACUC-20230327-20). A chronic atrophic gastritis (CAG) model was established using N-methyl-N′-nitro-N-nitrosoguanidine (MNNG) combined with ranitidine and irregular feeding. Briefly, mice received MNNG (150 μg/mL; YCKJ-YK-M706, Psaitong, China) by gavage every other day, were fed chow containing ranitidine (0.05%; R838252, Macklin, Shanghai, China), and were subjected to irregular feeding to facilitate disease progression. The total modeling period lasted 22 weeks. After 4 weeks of model induction, mice in the modeling cohort were randomized into the following groups: model, folic acid (FA, positive control), and HJSS-L/M/H groups. Treatments were then administered by gavage for 18 consecutive weeks. HJSS-L, HJSS-M, and HJSS-H were administered at doses of 0.348, 0.698, and 1.396 g/kg/day, respectively, and the FA group received folic acid at 1.03 mg/kg/day. Mice in the control and model groups received an equal volume of saline. At the endpoint, mice were anesthetized with 1% sodium pentobarbital (50 mg/kg, intraperitoneal injection) and euthanized by cervical dislocation under deep anesthesia. Blood, stomach tissues, and major organs (liver, spleen, and kidney) were subsequently collected for further analyses.

### 2.3 Preparation of HJSS-medicated serum

Healthy SD rats (4 weeks old, 180–220 g) were randomly assigned to a blank serum group (equal volume of saline) or an HJSS-medicated serum group. Rats received gavage twice daily for 3 consecutive days. One hour after the last administration, blood was collected via cardiac puncture, allowed to clot at room temperature, and centrifuged at 4 °C to obtain serum. Serum was heat-inactivated at 56 °C for 30 min, sterilized through a 0.22-μm filter, aliquoted, and stored at −80 °C. For cell experiments, medicated serum was added to the culture medium at the indicated volume fractions (5%, 10%, and 15%, v/v). The total serum content was kept constant among groups; NC and model groups received the same proportion of blank serum. The pharmacologically active constituents in HJSS-medicated serum were not quantitatively determined in the present study. The medicated serum was used as an *in vitro* intervention to approximate the post-administration serum milieu after oral HJSS exposure, while the chemical consistency of the HJSS extract itself was assessed by LC–MS fingerprinting.

### 2.4 Histology, immunohistochemistry, and immunofluorescence

Gastric tissues were fixed in 4% paraformaldehyde, paraffin-embedded, and sectioned (4–5 μm). Hematoxylin and eosin (H&E) staining was performed to evaluate mucosal architecture, inflammatory infiltration, and atrophic changes. Alcian blue–periodic acid–Schiff (AB–PAS) staining was used to assess mucus layer–associated glycoproteins/mucins. The severity of gastric mucosal atrophy was scored according to predefined criteria. Immunohistochemistry (IHC) was performed to detect ATP4B, TNF-α, CFTR, ZO-1, and additional proteins in gastric tissue. Briefly, sections were deparaffinized, rehydrated, subjected to antigen retrieval, blocked, and incubated with primary antibodies overnight at 4 °C, followed by appropriate secondary antibodies, DAB development, and hematoxylin counterstaining. Immunofluorescence (IF) was conducted to assess CFTR/ZO-1 colocalization in gastric tissue and p65 nuclear localization in GES-1 cells. Images were captured using a fluorescence microscope, and signal intensity was quantified using ImageJ. Antibody information is provided in **Supplementary Table 1**.

### 2.5 Serum cytokines and gastric function indices

Serum IL-6, IL-1β, IL-10, IFN-γ, and TNF-α were quantified using a cytometric bead array (CBA) kit (BK-CBA-4-01245, Absin, Shanghai, China) according to the manufacturer’s instructions. Serum pepsinogen I (PGI), pepsinogen II (PGII), and gastrin-17 (G-17) were measured using ELISA kits (AF0066-MA, AiFang, China). Standard curves and quality controls were included. Each sample was measured in at least triplicate.

### 2.6 Cell culture and treatments

The human gastric epithelial cell line GES-1 was obtained from Zhejiang Meisen Cell Technology Co., Ltd. (Zhejiang, China). Cells were cultured in RPMI-1640 medium supplemented with 10% fetal bovine serum and 1% penicillin–streptomycin at 37 °C in a humidified incubator with 5% CO_2_. Cells in the logarithmic growth phase were used for subsequent experiments. To establish an injury model, GES-1 cells were treated with graded concentrations of MNNG for 24 h to determine an appropriate modeling concentration. For mechanistic validation, the CFTR inhibitor CFTRinh-172 (C2992, Merck, Darmstadt, Germany) was used to explore the involvement of CFTR-associated regulation in HJSS-related epithelial barrier protection, and the NF-κB inhibitor BAY 11-7082 (HY-13453, MedChemExpress, Monmouth Junction, NJ, USA) was used to evaluate NF-κB involvement and its association with CFTR regulation.

### 2.7 Cell viability, morphology, and apoptosis assays

Cell viability was measured using the Cell Counting Kit-8 (CCK-8) assay. After treatment, CCK-8 reagent (C0039, Beyotime, Shanghai, China) was added and incubated at 37 °C for 1 h, and absorbance was measured at 450 nm. Data were normalized to the corresponding control group. Cell morphology was observed under an inverted microscope. Nuclear morphological changes associated with apoptosis were evaluated using Hoechst 33342 staining (C1026, Beyotime). Apoptosis was quantified by flow cytometry using an Annexin V-FITC/PI apoptosis detection kit (C1383L, Beyotime) according to the manufacturer’s protocol, and the percentage of apoptotic cells was analyzed.

### 2.8 Western blotting and nuclear/cytoplasmic fractionation

Total proteins from gastric tissues or GES-1 cells were extracted using RIPA lysis buffer supplemented with protease and phosphatase inhibitors. Protein concentration was determined by a BCA assay. Equal amounts of protein were separated by SDS-PAGE and transferred onto PVDF membranes. Membranes were blocked and incubated with primary antibodies overnight at 4 °C, followed by HRP-conjugated secondary antibodies. Protein bands were visualized using an enhanced chemiluminescence system (ECL; P0018S, Beyotime) and quantified with ImageJ. Band intensities were normalized to the corresponding loading controls. To assess NF-κB activation, nuclear and cytoplasmic proteins were isolated using a commercial extraction kit (P0028, Beyotime). p65 distribution was analyzed by Western blot; GAPDH was used as a cytoplasmic marker and Histone H3 as a nuclear marker. Antibody details are listed in **Supplementary Table 1**.

### 2.9 Transcriptomic sequencing analysis

Gastric tissues from the control, model, and HJSS-medium groups (n = 3 per group) were subjected to RNA sequencing. Total RNA was extracted using TRIzol reagent, and RNA quantity and quality were assessed prior to library construction. Sequencing and bioinformatics analyses were performed by Shanghai Majorbio Bio-Pharm Technology Co., Ltd. (Shanghai, China). After quality control, clean reads were mapped to the reference genome and quantified. Differentially expressed genes (DEGs) were identified using the criteria |log_2_ fold change| > 1 and *P* < 0.05. Principal component analysis and clustering were performed. Gene Ontology (GO) and Kyoto Encyclopedia of Genes and Genomes (KEGG) enrichment analyses were conducted. Protein–protein interaction (PPI) networks were constructed using the STRING database and visualized in Cytoscape to identify key nodes.

### 2.10 Network pharmacology analysis

Active compounds of HJSS were retrieved from the Traditional Chinese Medicine Systems Pharmacology (TCMSP) database using oral bioavailability (OB) > 30% and drug-likeness (DL) > 0.18 as screening criteria. CAG-related targets were collected from GeneCards, OMIM, and additional databases, standardized, and intersected with HJSS-derived targets to obtain candidate targets. An “HJSS–compound–target” network was constructed, and a PPI network was generated using STRING. Core targets were identified based on topological parameters. GO and KEGG enrichment analyses were performed to predict potential pathways. Network construction and visualization were conducted mainly using Cytoscape.

### 2.11 Statistical analysis

Data are presented as mean ± SEM. Statistical analyses were performed using GraphPad Prism 8.0. For normally distributed data with homogeneous variances, one-way analysis of variance (ANOVA) was used. Welch’s ANOVA or nonparametric tests were applied when assumptions were not met. A two-tailed *P* < 0.05 was considered statistically significant.

## 3 Results

### 3.1 Chemical characterization of HJSS by LC–MS fingerprinting

**Figure 1:**
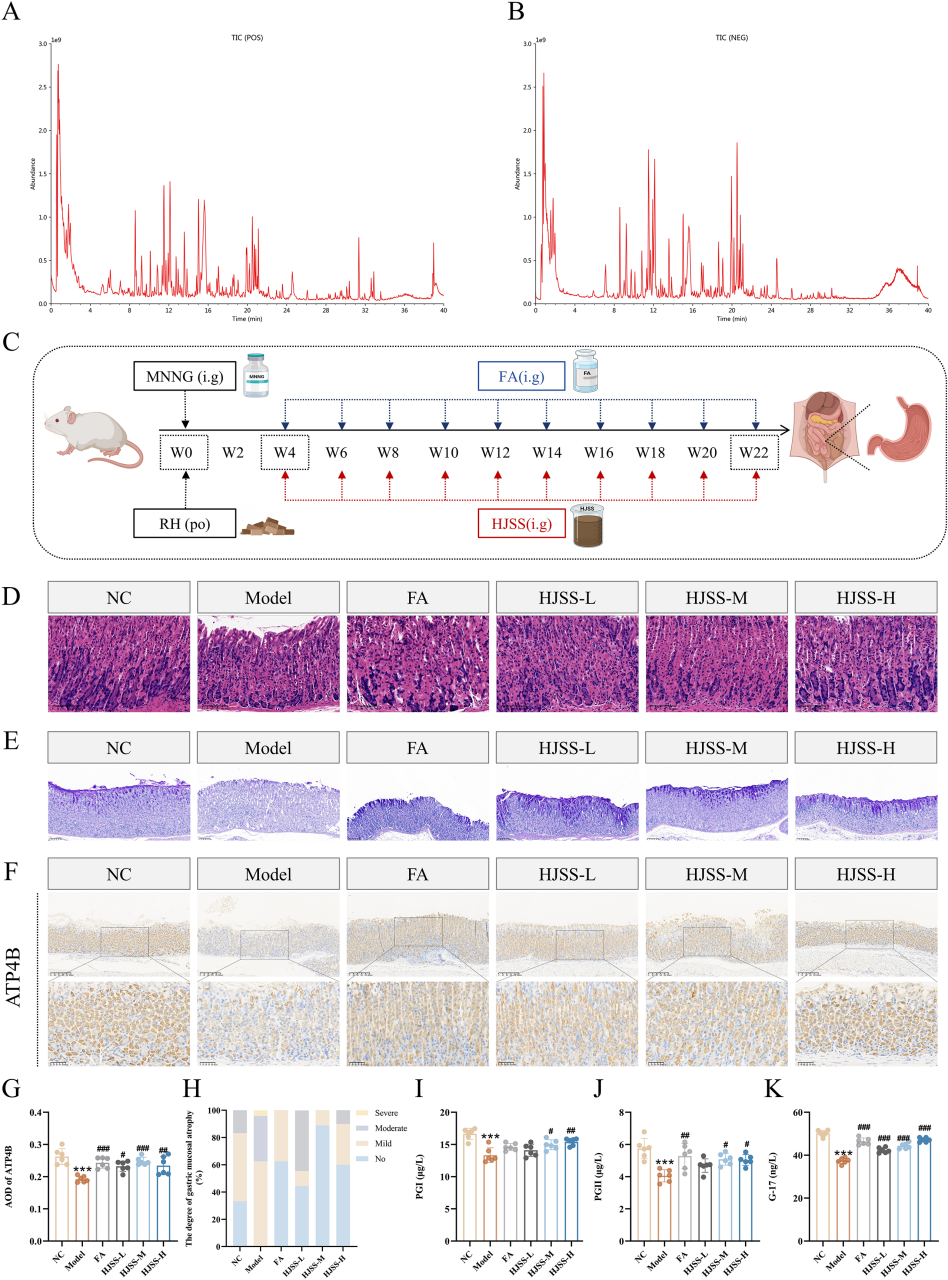
Chemical profiling of HJSS and its protective effects on gastric pathology and function in CAG. **(A)** TIC in positive ion mode. **(B)** TIC in negative ion mode. **(C)** Schematic illustration of animal modeling and treatment schedule. **(D)** Representative H&E-stained gastric sections. **(E)** Representative AB–PAS (Alcian blue–PAS) staining of gastric tissues. **(F, G)** Representative IHC images and quantitative analysis of ATP4B in gastric tissues. **(H)** Distribution of gastric mucosal atrophy severity. **(I–K)** Quantification of gastric function indices: PGI, PGII, and gastrin-17 (G-17). Data are presented as mean ± SEM (n = 6 mice per group). ***P < 0.001 vs. NC group; ^#^*P* < 0.05, ^##^*P* < 0.01, ^###^*P* < 0.001 vs. Model group.

To provide quality-control evidence for the herbal preparation used in this study, HJSS was characterized by LC–MS fingerprinting. Total ion chromatograms obtained in positive and negative ion modes displayed stable and reproducible peak patterns, supporting the chemical consistency of the aqueous extract across batches (**Figures 1A, B**). In addition, representative marker compounds derived from the component herbs were identified and are listed in **Table 2**. These data establish the analytical basis for the subsequent pharmacological evaluation of HJSS in CAG.

**Table 2:** Representative QC marker compounds for HJSS decoction.

Herb (genus)	Marker compound	Ion mode	m/z	RT (min)	Rel. abundance (%)	Mass error (ppm)
Astragalus	Methylnissolin-3-O-glucoside	POS	480.18681	20.862	0.332980333	0.812185
Astragalus	Raffinose	NEG	549.16815	0.851	0.306604193	1.675264
Astragalus	Astragaloside II	NEG	871.47125	26.062	0.123418532	1.813029
Astragalus	Isomucronulatol 7-O-glucoside	POS	487.15724	21.008	0.093474653	-0.472127
Lonicera	Secologanic acid	NEG	373.11442	12.124	10.38564657	1.072058
Lonicera	Secologanin	POS	411.12595	15.597	4.864567632	-0.510792
Lonicera	Isochlorogenic acid C	NEG	515.11916	19.951	3.997849143	-0.660041
Lonicera	Sweroside	NEG	403.12413	13.579	3.602659289	-1.11628
Scrophularia	N-Feruloyltyramine	POS	336.12259	20.719	2.233900912	5.83124
Scrophularia	Harpagide	NEG	363.13012	8.555	1.125970023	1.239227
Scrophularia	Sibirioside A	POS	495.14786	17.376	0.193869295	1.151173
Scrophularia	Neodiosmin	NEG	607.16846	20.03	0.012208637	2.66813
Salvia	Salvianolic acid Y	NEG	717.14774	20.56	10.74086966	2.272898
Salvia	Rosmarinic acid	NEG	359.0777	19.966	2.763496458	1.281061
Salvia	Cryptotanshinone	POS	319.13086	31.366	2.072624427	1.253406
Salvia	Danshensu	NEG	197.04518	7.116	1.797817865	-1.877738
Angelica	Levistolide A	POS	403.18825	32.741	0.178552207	0.669663
Angelica	Robinetin	POS	303.05017	17.626	0.096536383	0.791949
Angelica	3’-Methoxypuerarin	NEG	445.11427	14.223	0.020699384	0.561654
Angelica	Senkyunolide G	POS	191.10695	23.582	0.015184803	-1.517473
Actinidia	Kaempferol-3-O-glucorhamnoside	NEG	593.15115	17.601	0.049427447	-0.067436
Actinidia	Gallocatechin	NEG	611.13941	7.839	0.022104694	-1.996267
Actinidia	19alpha-Hydroxyasiatic acid	POS	487.34114	23.307	0.019413194	-2.564932
Actinidia	Quercetin	NEG	301.03574	21.05	0.016321393	1.195873
Glycyrrhiza	Licoisoflavone A	NEG	353.10336	28.78	0.573316367	0.84961
Glycyrrhiza	Licorice-saponin H2	NEG	821.39959	26.098	0.47376837	3.749712
Glycyrrhiza	Licorice saponin G2	POS	839.40725	23.307	0.34243028	1.512975
Glycyrrhiza	glycyrrhizinate	POS	845.39349	24.601	0.254991171	0.579612

### 3.2 HJSS alleviated histopathological injury and gastric functional impairment in MNNG-induced CAG

The overall experimental workflow for MNNG-induced CAG modeling and drug intervention is shown in **Figure 1C**. To evaluate the therapeutic effects of HJSS on experimental CAG, gastric histopathology and functional indices were first assessed in MNNG-treated mice. H&E staining showed that MNNG induced typical pathological features of CAG, including disorganized glandular architecture, inflammatory infiltration, mucosal thinning, and glandular atrophy. These histopathological alterations were markedly alleviated by HJSS treatment, indicating that HJSS mitigated structural injury in the gastric mucosa (**Figure 1D**).

AB–PAS staining further demonstrated that MNNG caused evident mucus-associated abnormalities, whereas HJSS partially restored mucosal staining intensity and histochemical appearance (**Figure 1E**). In parallel, ATP4B expression was markedly reduced in the model group and was restored to varying extents after HJSS administration (**Figures 1F, G**), suggesting improvement not only of mucosal structure but also of function-related epithelial impairment. Quantification of mucosal atrophy further confirmed that HJSS reduced the severity of gastric mucosal atrophy, with the medium-dose group showing the most pronounced improvement (**Figure 1H**).

These histological benefits were further supported by serum gastric functional indices. MNNG-induced CAG disrupted the levels of PGI, PGII, and G-17, whereas HJSS significantly ameliorated these abnormalities (**Figures 1I–K**). In addition, organ indices of the liver, spleen, and kidney showed no overt adverse shift attributable to HJSS under the present dosing conditions (**Supplementary Figures 1A–C**), supporting the general tolerability of the intervention. Collectively, these findings indicate that HJSS alleviated both the histopathological and functional manifestations of MNNG-induced CAG.

### 3.3 HJSS mitigated inflammatory burden and apoptosis-associated epithelial injury in CAG

**Figure 2:**
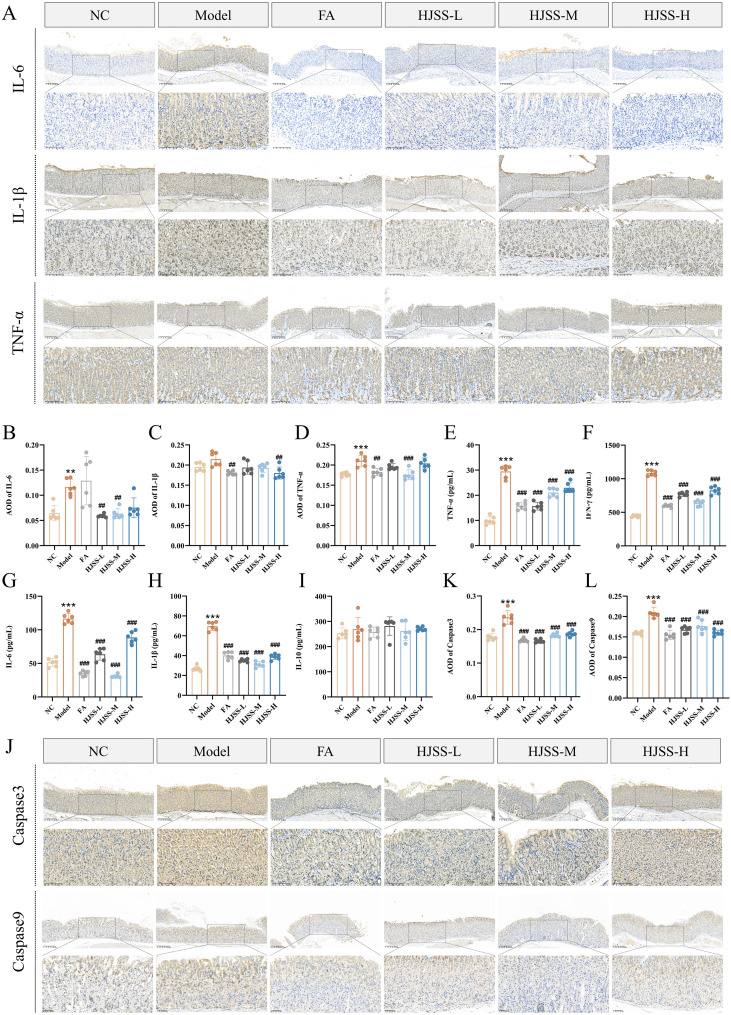
HJSS attenuates inflammatory burden and epithelial injury in CAG. **(A–D)** Representative IHC images and quantitative analysis of IL-6, IL-1β, and TNF-α in gastric tissues. **(E–I)** Quantification of serum cytokines: IL-6, IL-1β, IL-10, IFN-γ, and TNF-α. **(J–L)** Representative IHC images and quantitative analysis of Caspase-3, and Caspase-9 in gastric tissues. Data are presented as mean ± SEM (n = 6 mice per group). **P < 0.01, ***P < 0.001 vs. NC group; ^##^*P* < 0.01, ^###^*P* < 0.001 vs. Model group.

Because persistent inflammatory activation is a key feature of CAG progression, we next examined whether HJSS modulated inflammatory burden in gastric tissues and circulation. Immunohistochemical analysis showed that IL-6, IL-1β, and TNF-α were markedly elevated in the gastric mucosa of model mice, indicating sustained local inflammatory activation under MNNG exposure (**Figures 2A–D**). HJSS treatment significantly reduced the expression of these pro-inflammatory cytokines, supporting an inhibitory effect on the local inflammatory response in CAG.

We then evaluated whether this effect was also reflected systemically. Serum cytokine analysis showed that the inflammatory profile was disrupted in CAG mice, whereas HJSS partially corrected these abnormalities (**Figures 2E–I**). The overall consistency between gastric mucosal cytokine reduction and systemic inflammatory improvement indicates that the anti-inflammatory action of HJSS was not confined to a single tissue compartment.

To further assess apoptosis-associated epithelial injury *in situ*, Caspase-3 and Caspase-9 were examined in gastric tissues by immunohistochemistry, which preserves mucosal architecture and localization information. MNNG markedly increased Caspase-3- and Caspase-9-associated apoptotic signaling, whereas HJSS attenuated these alterations (**Figures 2J–L**). These findings indicate that HJSS mitigated the inflammatory burden of CAG and concomitantly reduced apoptosis-associated epithelial injury *in vivo*.

### 3.4 Integrated transcriptomic and network pharmacology analyses guided the focus on TNF/CFTR-associated inflammation–barrier crosstalk

**Figure 3:**
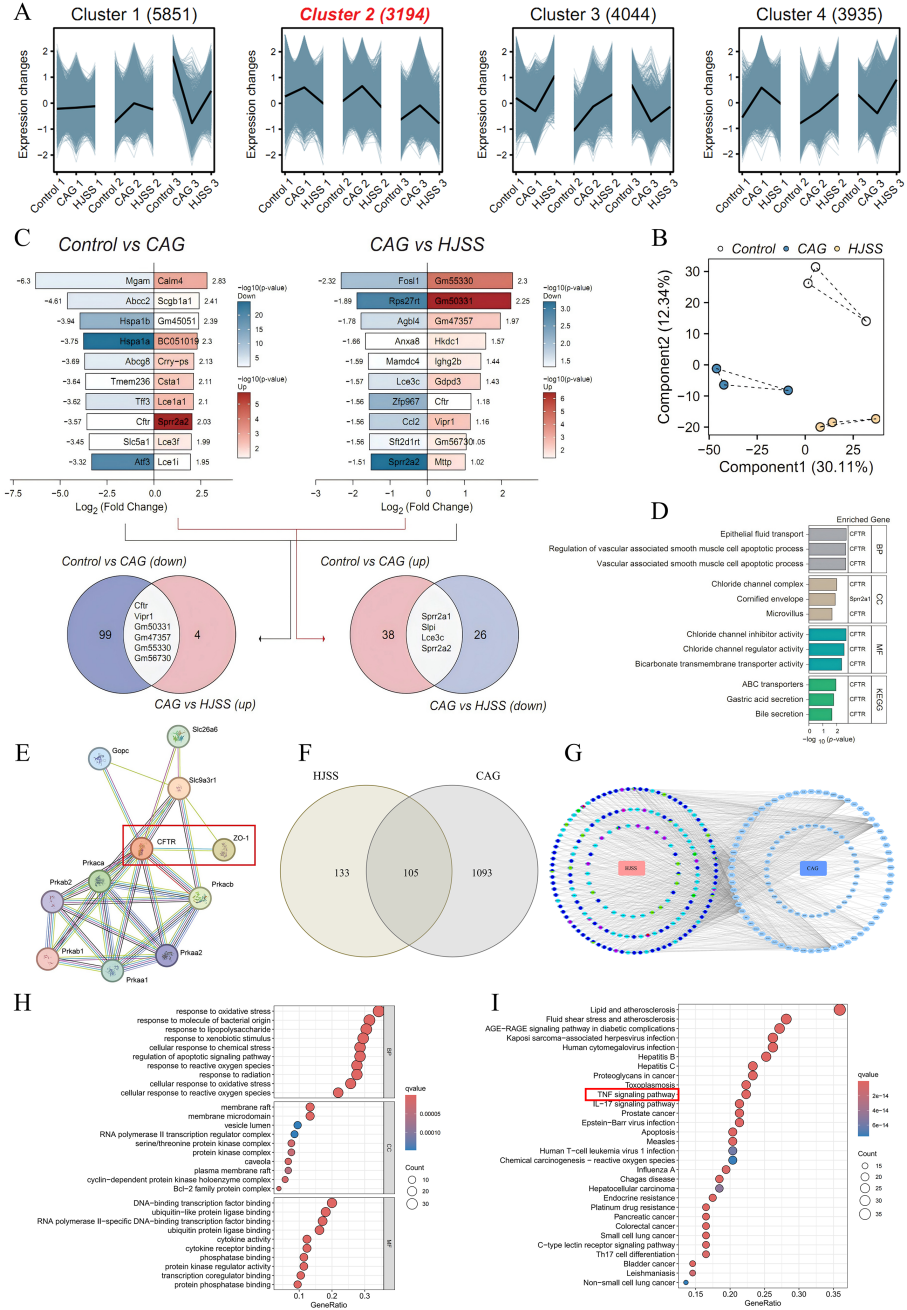
Integrated transcriptomic and network pharmacology analyses identify TNF/CFTR-associated inflammation–barrier crosstalk in CAG. **(A)** Time-series clustering analysis of differentially expressed genes. **(B)** Principal component analysis (PCA). **(C)** Differential gene visualization and Venn analysis. **(D)** GO/KEGG enrichment analysis. **(E)** PPI network analysis showing key molecular nodes and their interactions. **(F)** Venn diagram of overlapping targets between HJSS and CAG. **(G)** HJSS–active compound–target network. **(H, I)** GO/KEGG enrichment analysis. RNA-seq was performed with n = 3 biological replicates per group.

To further delineate the molecular events associated with HJSS intervention, transcriptomic sequencing was performed using gastric mucosal tissues from the control, model, and HJSS-treated groups. Time-series clustering analysis of differentially expressed genes identified clusters showing relatively consistent disease-associated trends, with representative concordant patterns highlighted in red (**Figure 3A**). Principal component analysis further demonstrated clear separation among groups at the transcriptomic level, indicating distinct global transcriptional profiles after MNNG injury and HJSS intervention (**Figure 3B**). Differential expression analysis and intersection screening were then used to identify genes that were significantly altered in the model group but showed opposite trends after HJSS treatment (**Figure 3C**).

GO and KEGG enrichment analyses suggested that these differentially expressed genes were mainly involved in epithelial homeostasis- and barrier-related processes, with CFTR appearing in multiple enriched terms (**Figure 3D**). PPI network analysis further identified CFTR as a candidate barrier-related node and suggested a potential interaction context linking CFTR to the barrier-associated protein ZO-1 (**Figure 3E**). These transcriptomic and PPI findings provided the basis for selecting CFTR and ZO-1 as representative barrier-associated molecules for subsequent experimental validation.

To complement the transcriptomic analysis from a formula–target perspective, network pharmacology was performed for HJSS and CAG. Overlapping targets were identified between HJSS-derived targets and CAG-related targets, and enrichment analysis indicated that apoptosis- and inflammation-related pathways were prominently represented, with TNF signaling emerging as a major candidate pathway (**Figures 3F–I**). Thus, transcriptomic analysis primarily highlighted CFTR-associated epithelial barrier regulation, whereas network pharmacology prioritized TNF-related inflammatory signaling. The convergence of these two analytical layers provided the rationale for subsequent validation of CFTR/ZO-1-associated barrier molecules and TNF/NF-κB signaling.

### 3.5 HJSS promoted recovery of gastric mucosal barrier-associated molecules *in vivo*

**Figure 4:**
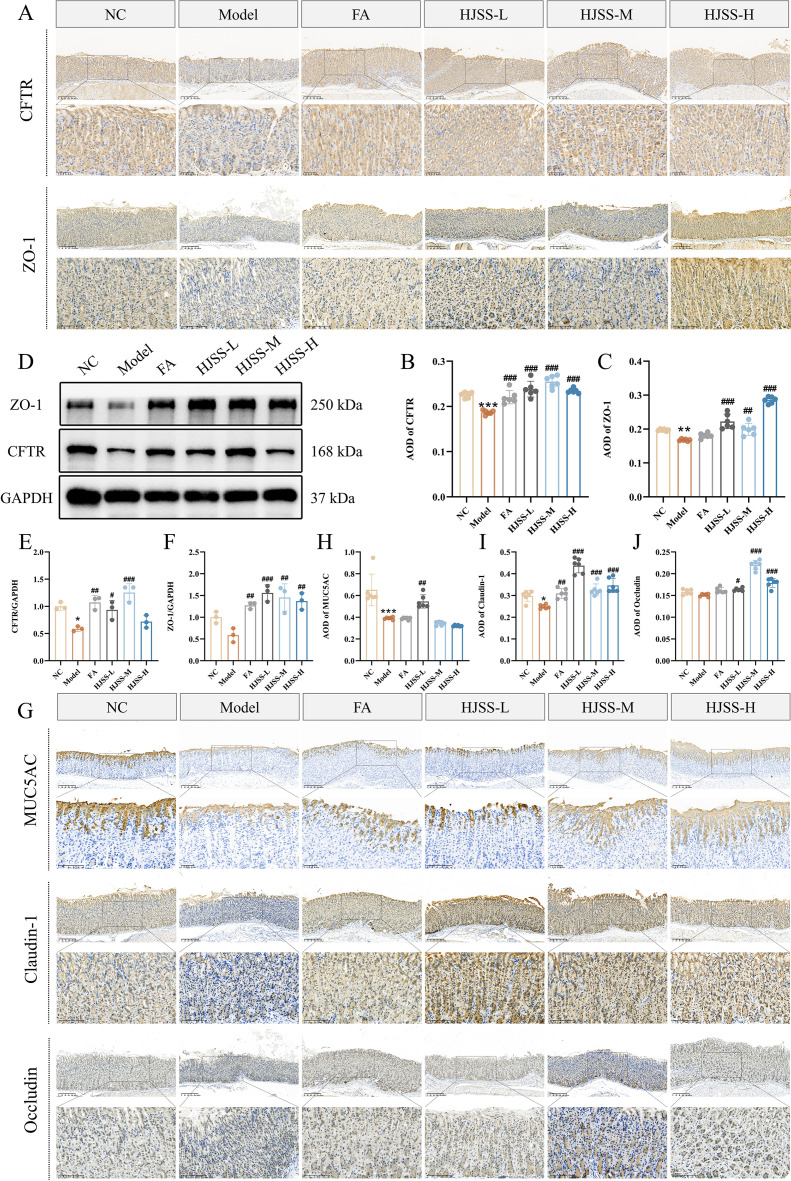
HJSS promotes recovery of gastric mucosal barrier-associated molecules *in vivo*. **(A–C)** Representative IHC images and quantitative analysis of CFTR and ZO-1 in gastric tissues. **(D–F)** Representative Western blot bands and quantitative analysis of CFTR and ZO-1 in gastric tissues. **(G–J)** Representative IHC images and quantitative analysis of MUC5AC, Occludin, and Claudin-1 in gastric tissues. Data are presented as mean ± SEM (IHC: n = 6 mice per group; WB: n = 3 independent mouse samples per group). *P < 0.05, ***P < 0.001 vs. NC group; ^#^*P* < 0.05, ^##^*P* < 0.01, ^###^*P* < 0.001 vs. Model group.

Based on the transcriptomic prioritization of CFTR-associated barrier regulation and the PPI-indicated CFTR–ZO-1 interaction context, we next examined whether HJSS modulated gastric mucosal barrier-associated molecules *in vivo*. CFTR expression was markedly reduced in the gastric mucosa of model mice, as demonstrated by both immunohistochemistry and western blotting, whereas HJSS significantly restored CFTR expression. A similar pattern was observed for ZO-1, which was disrupted in CAG mice but partially recovered after HJSS treatment (**Figures 4A–F**).

**Figure 5:**
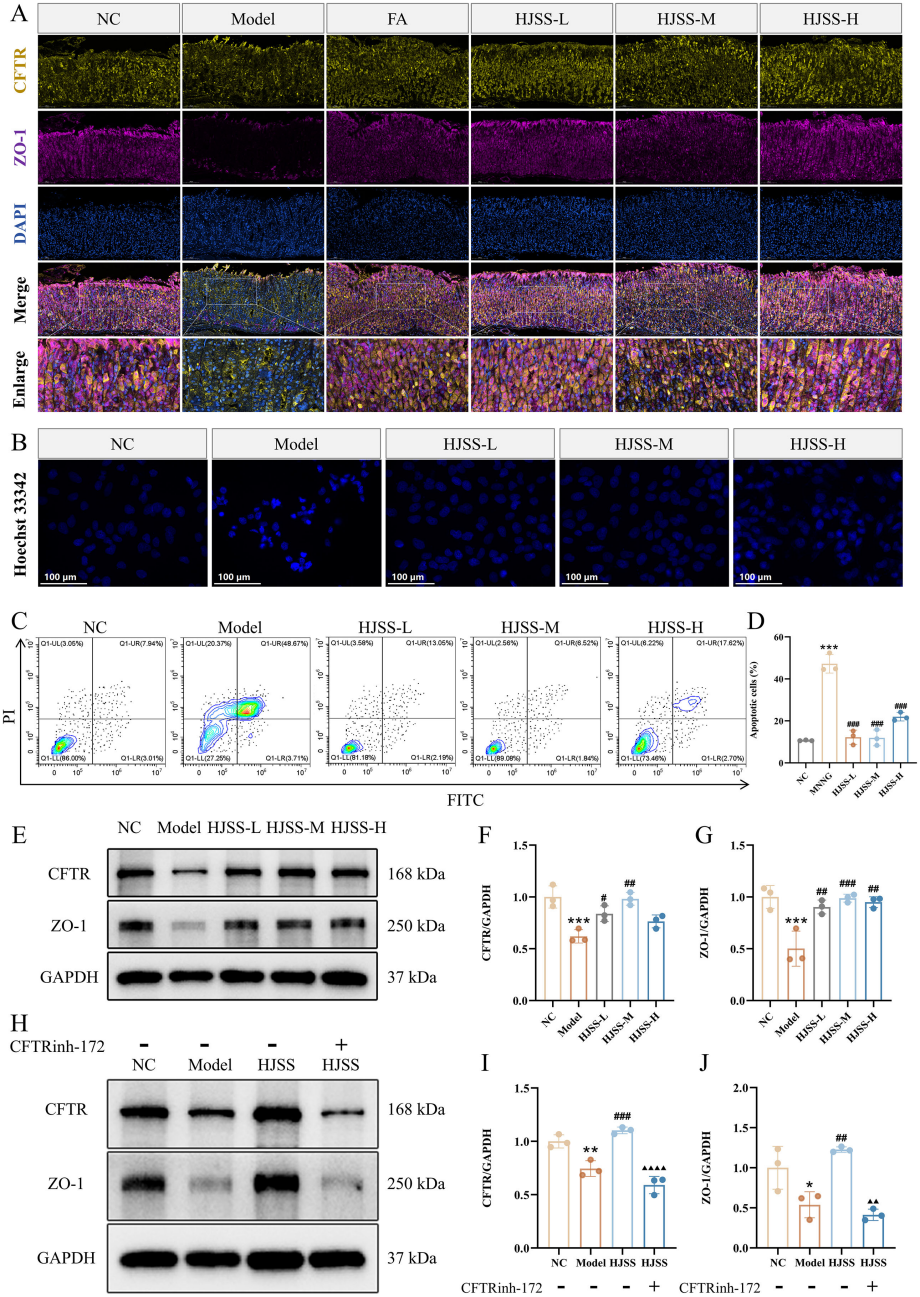
HJSS protects against MNNG-induced epithelial injury and supports CFTR-associated barrier regulation. **(A)** Representative IF images showing colocalization of CFTR and ZO-1 in gastric tissues. **(B)** Representative Hoechst 33342 staining images. **(C, D)** Representative flow cytometry plots and quantitative analysis of apoptosis. **(E–G)** Western blot analysis and quantitative assessment of CFTR and ZO-1 expression in GES-1 cells. **(H–J)** Western blot analysis and quantitative assessment of CFTR and ZO-1 expression in GES-1 cells treated with CFTRinh-172. Data are presented as mean ± SEM (IF: n = 6 mice per group; flow cytometry and WB: n = 3 independent experiments). *P < 0.05, **P < 0.01, ***P < 0.001 vs. NC group; ^#^*P* < 0.05, ^##^*P* < 0.01, ^###^*P* < 0.001 vs. Model group; ^▲▲^*P* < 0.01, ^▲▲▲▲^*P* < 0.001, HJSS + CFTRinh-172 vs. HJSS.

To further assess the *in situ* relationship between barrier-associated molecules, immunofluorescence staining of gastric tissues was performed. The model group showed impaired CFTR and ZO-1 signals, whereas HJSS enhanced the expression of both proteins and supported their coordinated recovery within the gastric mucosa (**Figure 5A**). This spatial evidence was consistent with the bulk protein data and further supported recovery of barrier-associated molecular organization *in vivo*.

We next examined additional components of gastric barrier organization, including MUC5AC, Occludin, and Claudin-1. MNNG-induced CAG markedly reduced the expression of these molecules, indicating concurrent impairment of mucus-associated protection and epithelial junctional integrity (**Figures 4G–J**). HJSS significantly restored their expression, suggesting that its protective effect was not restricted to a single marker but involved coordinated recovery of multiple barrier-associated elements. Collectively, these findings indicate that HJSS promoted the recovery of gastric mucosal barrier-associated molecular features *in vivo*, thereby providing a molecular basis for its histological and functional benefits.

### 3.6 HJSS protected GES-1 cells against MNNG-induced epithelial injury

To determine whether HJSS directly protects gastric epithelial cells under injurious conditions, an MNNG-induced GES-1 cell injury model was established and treated with HJSS-medicated serum. Preliminary concentration-screening experiments were performed to identify suitable concentrations of MNNG and HJSS-medicated serum for subsequent assays (**Supplementary Figures 1D–F**).

Under the selected conditions, MNNG markedly reduced cell viability and induced obvious morphological damage in GES-1 cells, whereas HJSS-medicated serum significantly improved cell status and alleviated injury-associated morphological alterations (**Supplementary Figure 1G**). Hoechst 33342 staining further showed that MNNG induced characteristic nuclear condensation and apoptotic morphology, which were attenuated by HJSS-medicated serum (**Figure 5B**). Flow cytometric analysis consistently demonstrated that HJSS significantly reduced the apoptotic rate of MNNG-injured GES-1 cells (**Figures 5C, D**).

These findings indicate that HJSS-medicated serum protected GES-1 cells against MNNG-induced epithelial injury and attenuated apoptosis *in vitro*. The consistency between the cellular and *in vivo* results supports the view that epithelial protection constitutes an important component of the overall action of HJSS in CAG.

### 3.7 CFTR inhibition attenuated the HJSS-associated restoration of CFTR/ZO-1 expression in MNNG-injured GES-1 cells

To further assess whether CFTR-associated regulation is involved in the epithelial protective effect of HJSS, CFTR and ZO-1 expression were examined in MNNG-injured GES-1 cells with or without CFTR inhibition. Consistent with the *in vivo* findings, MNNG markedly reduced the expression of both CFTR and ZO-1, whereas HJSS-medicated serum partially restored these proteins (**Figures 5E–G**). This consistency between the animal and cell models supports the view that CFTR-associated barrier-related alterations are reproducible features of MNNG-induced epithelial injury rather than incidental tissue-level changes.

To further probe the functional relevance of this observation, cells were treated with the CFTR inhibitor CFTRinh-172. Under CFTR inhibition, the HJSS-associated restoration of CFTR and ZO-1 expression was significantly attenuated (**Figures 5H–J**). These findings provide pharmacological support for the involvement of CFTR-associated regulation in the barrier-protective effects of HJSS in injured gastric epithelial cells. However, because the present evidence is based mainly on expression changes and pharmacological inhibition, this result should not be interpreted as establishing a direct, exclusive, or phosphorylation-dependent CFTR activation mechanism. Additional molecular interactions are likely to contribute to the overall protective response.

### 3.8 HJSS was associated with suppression of TNF/NF-κB signaling and reduced p65 nuclear translocation

**Figure 6:**
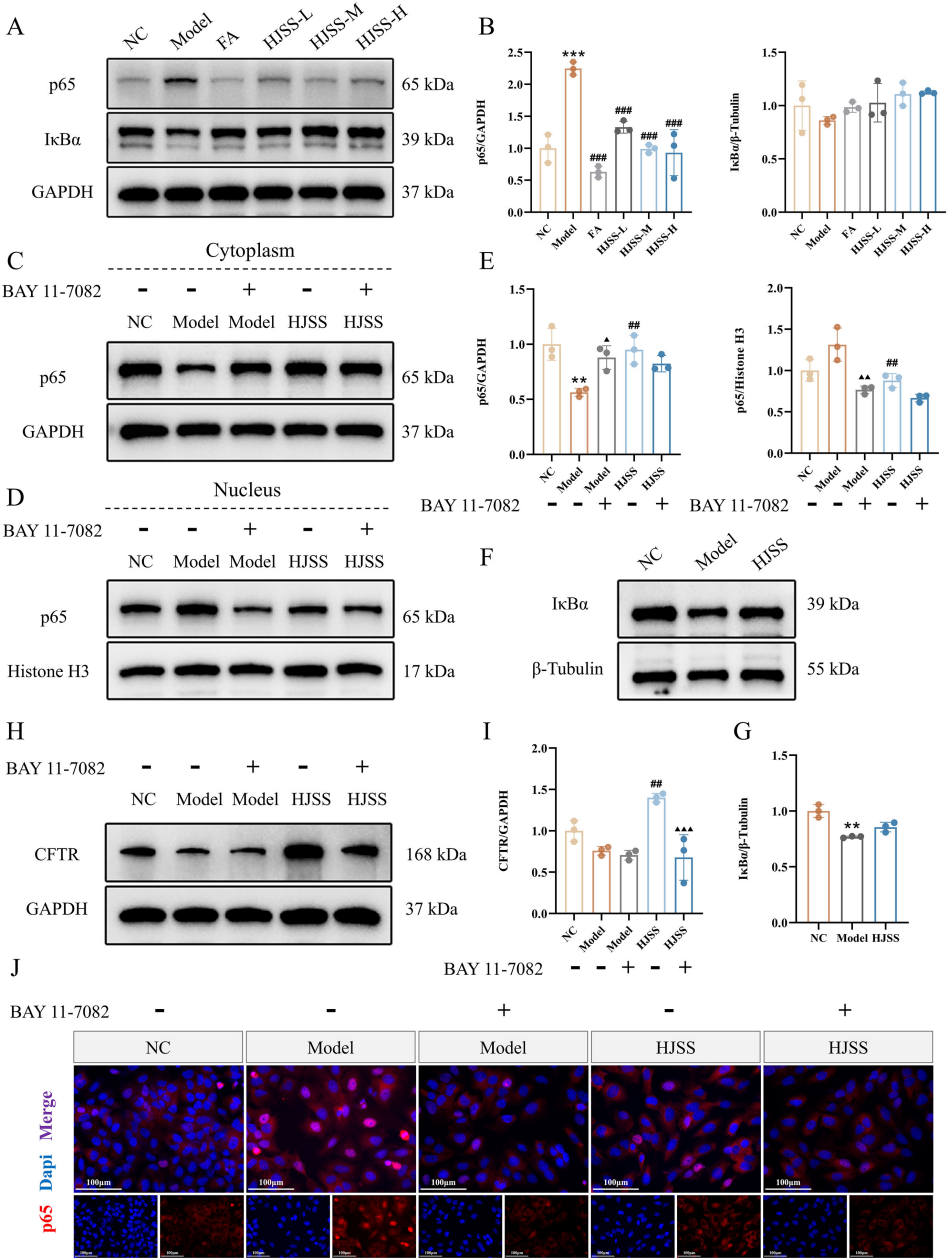
HJSS suppresses NF-κB activation and reduces p65 nuclear translocation. **(A, B)** Representative Western blot bands and quantitative assessment of p65 and IκBα expression in gastric tissues from mice. (**C–E)** Nuclear/cytoplasmic fractionation analysis of p65 distribution in GES-1 cells: GAPDH as the cytoplasmic loading control and Histone H3 as the nuclear loading control. **(F, G)** Western blot analysis and quantitative assessment of IκBα expression in GES-1 cells. **(H, I)** Western blot analysis and quantitative assessment of CFTR expression in GES-1 cells. **(J)** Representative IF images of p65. Data are presented as mean ± SEM (tissue WB: n = 3 independent mouse samples per group; cell WB and IF: n = 3 independent experiments). **P < 0.01, ***P < 0.001 vs. NC group; ^##^*P* < 0.01, ^###^*P* < 0.001 vs. Model group; ^▲^*P* < 0.05, ^▲▲^*P* < 0.01, Model + BAY 11–7082 vs. Model; ^▲▲▲^*P* < 0.001, HJSS + BAY 11–7082 vs. HJSS.

Because the integrated analyses highlighted TNF-related signaling and the *in vivo* data demonstrated substantial anti-inflammatory activity, we next explored whether NF-κB activation is linked to the protective effects of HJSS. In gastric tissues from model mice, p65 expression was elevated whereas IκBα expression was reduced, indicating activation of inflammatory signaling. HJSS treatment reversed these changes (**Figures 6A, B**). To further strengthen the evidence for NF-κB activation, phosphorylated p65 was examined as a supplementary activation marker. MNNG-induced CAG increased the p-p65/total p65 ratio in gastric tissues, whereas HJSS treatment reduced p65 phosphorylation (**Supplementary Figures 1J, K**), further supporting suppression of NF-κB activation *in vivo*. In MNNG-injured GES-1 cells, nuclear/cytoplasmic fractionation further showed enhanced nuclear accumulation of p65, a hallmark of NF-κB activation, while HJSS-medicated serum markedly reduced p65 nuclear translocation (**Figures 6C–E**). Consistently, MNNG increased the p-p65/total p65 ratio in GES-1 cells, whereas HJSS-medicated serum reduced p65 phosphorylation (**Supplementary Figures 1H, I**), in line with the observed decrease in p65 nuclear translocation.

**Figure 7:**
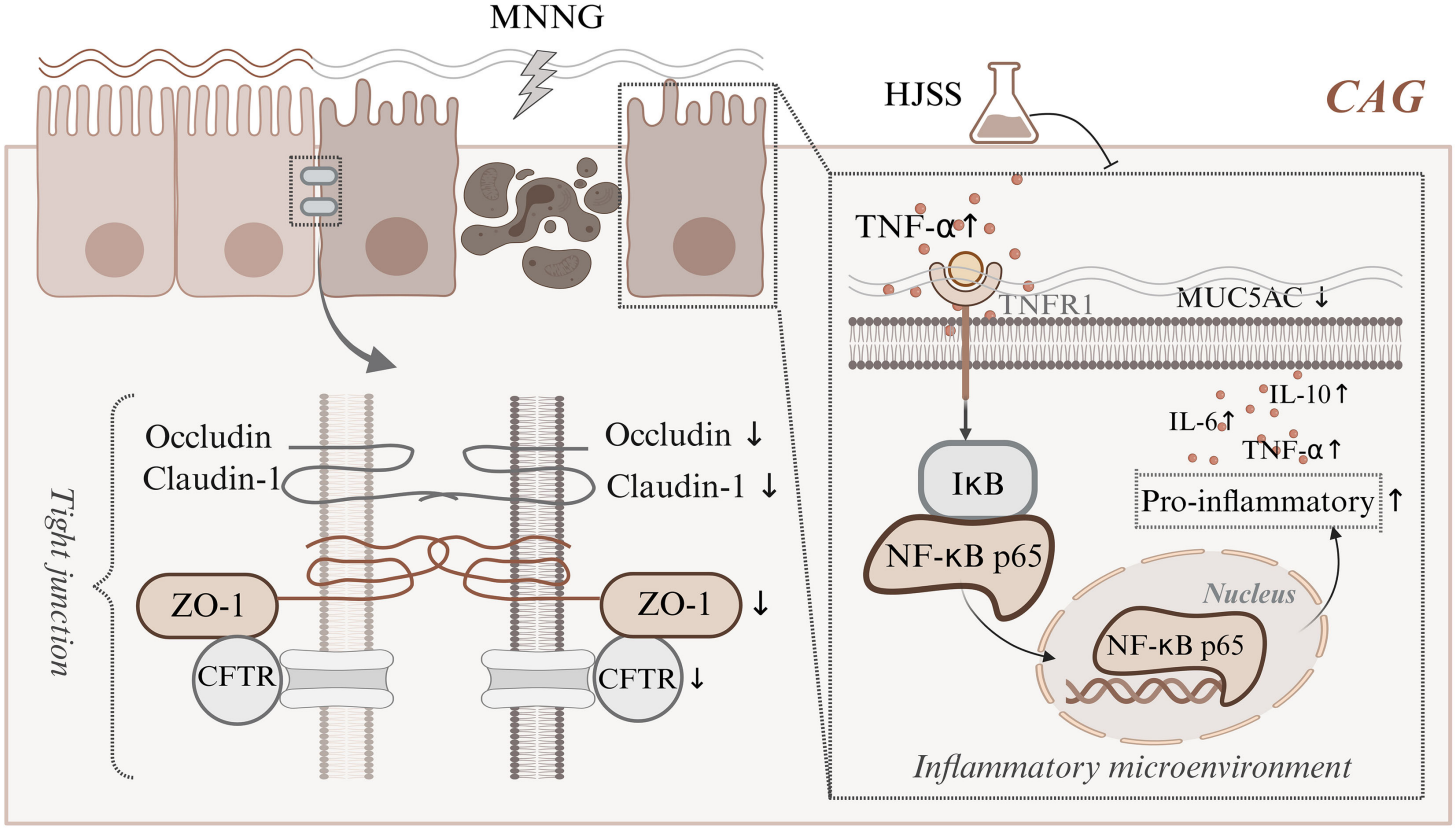
Proposed working model of HJSS in chronic atrophic gastritis.

To further evaluate the relevance of NF-κB activity, BAY 11–7082 was applied as a pharmacological inhibitor. NF-κB inhibition altered p65 (**Figures 6C–E**), IκBα (**Figures 6F, G**) and CFTR (**Figures 6H, I**) expression patterns in MNNG-injured cells, supporting an association between NF-κB activity and CFTR-associated epithelial changes. IF further confirmed the changes in p65 nuclear localization: MNNG enhanced nuclear p65 signals, whereas both HJSS and BAY 11–7082 reduced nuclear localization of p65, consistent with the fractionation results (**Figure 6J**). Thus, the TNF/NF-κB pathway appears to function as a signaling context within which barrier-associated molecular recovery occurs. Overall, these data support the interpretation that HJSS alleviates CAG through coordinated attenuation of inflammatory injury and recovery of barrier-associated molecular features, with TNF/NF-κB signaling and CFTR-associated regulation forming part of the mechanistic framework linking these two processes. A proposed working model summarizing the inflammation–barrier mechanism of HJSS in CAG is shown in **Figure 7**.

## 4 Discussion

This study supports the view that chronic atrophic gastritis (CAG) should be interpreted not only as a state of persistent mucosal inflammation, but also as a disorder involving impaired gastric mucosal barrier-associated integrity. In the present model, MNNG-induced CAG was accompanied by structural injury, functional impairment, increased inflammatory burden, apoptosis-associated epithelial damage, and coordinated disruption of barrier-related molecules. HJSS alleviated these alterations at both histological and molecular levels. Taken together, these findings suggest that the protective action of HJSS is better understood within an inflammation–barrier framework than as a nonspecific anti-inflammatory effect alone. In CAG, barrier impairment is not merely a downstream consequence of tissue injury; it also facilitates sustained exposure to luminal irritants and inflammatory stimuli, thereby perpetuating mucosal vulnerability and pathological progression (23–25). In this context, interventions capable of concurrently dampening inflammatory signaling and preserving epithelial barrier organization may be particularly relevant to disease control (26, 27). This interpretation was also supported by the integrated analytical strategy used in this study. Transcriptomic profiling primarily pointed to epithelial barrier-related processes and CFTR-associated regulation, whereas network pharmacology highlighted TNF-related inflammatory signaling. These two analytical layers therefore provided complementary entry points for subsequent validation of CFTR/ZO-1-associated barrier molecules and TNF/NF-κB pathway activity.

Inflammation is a central driver of CAG progression, and the TNF-α/NF-κB axis represents a biologically plausible signaling hub linking inflammatory amplification to epithelial stress and tissue remodeling. As an upstream inflammatory cytokine, TNF-α activates NF-κB through canonical signaling, promoting IκBα degradation and p65 nuclear translocation, which in turn drives transcription of inflammatory mediators and stress-related genes (28–30). Sustained activation of this axis is therefore well positioned to maintain chronic inflammatory signaling and exacerbate epithelial injury during the progression from gastritis to precancerous lesions (31–33). In the present study, HJSS reduced TNF-α and other pro-inflammatory mediators, suppressed p65 activation and nuclear translocation, and partially restored IκBα expression. Moreover, the effects observed with BAY 11–7082 further supported the involvement of NF-κB activity in downstream molecular regulation under MNNG-induced injury (34). These observations indicate that inhibition of TNF/NF-κB signaling constitutes an important component of the protective action of HJSS and provides a mechanistic context for its anti-inflammatory effects.

Beyond inflammatory signaling itself, maintenance of epithelial barrier integrity also depends on transport and junctional regulatory systems. CFTR has classically been defined as a chloride/bicarbonate channel, but growing evidence indicates that it additionally participates in epithelial differentiation, junctional organization, and barrier regulation through interactions with scaffold proteins such as ZO-1 (15, 35). From a pathobiological perspective, CFTR dysfunction has been linked to epithelial fragility, altered mucus properties, sustained inflammatory signaling, and a microenvironment permissive to chronic injury and malignant progression (36–38). Although its relevance to CAG has remained insufficiently clarified, the present study showed that CFTR expression was reduced in MNNG-induced CAG and was restored by HJSS both *in vivo* and *in vitro*, accompanied by recovery of ZO-1 and additional barrier-associated molecules. Importantly, CFTRinh-172 attenuated the HJSS-associated restoration of CFTR/ZO-1 expression (39), providing pharmacological support for the involvement of CFTR-associated regulation in the barrier-protective effects of HJSS. These data do not establish CFTR as a direct or exclusive mechanistic target of HJSS. Rather, based on expression changes and pharmacological inhibition, they support the interpretation that CFTR-associated regulation is functionally relevant to the epithelial protective network engaged by HJSS.

The reciprocal amplification between inflammatory signaling and barrier disruption is increasingly recognized as an important biological basis for the progression of CAG toward intestinal metaplasia and more advanced precancerous lesions. Pro-inflammatory cytokines can promote the relocalization or downregulation of ZO-1, Occludin, Claudin-1, and related junctional molecules, thereby weakening mucosal integrity and increasing epithelial vulnerability. Conversely, barrier impairment facilitates sustained exposure to luminal irritants and inflammatory stimuli, further perpetuating chronic epithelial damage (40, 41). In the present study, activation of TNF/NF-κB signaling coincided with reduced expression of CFTR, ZO-1, MUC5AC, Occludin, and Claudin-1 in the model group, whereas HJSS concurrently suppressed the inflammatory axis and promoted recovery of multiple barrier-associated molecular features. When considered together with the effects of BAY 11–7082 on p65 nuclear translocation and CFTR-related changes, these findings support a coupled mechanism in which inflammatory control may favor CFTR-associated barrier-related molecular recovery, thereby facilitating junctional and mucus-layer repair.

From a broader perspective, the present study provides a relatively integrated *in vivo* and *in vitro* evidence chain linking inflammatory suppression, epithelial protection, and barrier-associated molecular recovery in experimental CAG. This is potentially relevant because strategies directed only toward symptom control or general anti-inflammatory activity may be insufficient to interrupt the self-reinforcing cycle of chronic mucosal injury. In contrast, barrier-oriented interventions may offer an additional mechanistic dimension for limiting disease persistence and possibly delaying progression toward more advanced precancerous lesions. In this sense, the present work may provide a useful mechanistic framework for understanding how multi-component herbal formulas act in chronic gastric mucosal injury, while also increasing its conceptual relevance to the Inflammation section of Frontiers in Immunology.

Nevertheless, several limitations should be acknowledged. First, the barrier-related conclusions are based mainly on barrier-associated molecular markers, including CFTR, ZO-1, Occludin, Claudin-1, and MUC5AC, together with AB–PAS staining, rather than direct functional assays of epithelial permeability. Therefore, these findings are interpreted as recovery of barrier-associated molecular features rather than definitive restoration of gastric mucosal barrier homeostasis. Second, although LC–MS fingerprinting was performed to characterize the HJSS extract, the absorbed or metabolized constituents in HJSS-medicated serum were not quantitatively determined. Third, the present data support CFTR-associated involvement in barrier-related molecular recovery but do not define phosphorylation-dependent CFTR activation or CFTR channel function.

## 5 Conclusion

HJSS alleviated MNNG-induced chronic atrophic gastritis by attenuating inflammatory injury and promoting the recovery of gastric mucosal barrier-associated molecular features. Its protective effects were associated with suppression of TNF/NF-κB signaling, reduced p65 nuclear translocation, and recovery of CFTR-associated barrier-related molecular expression. Collectively, the present findings support an inflammation–barrier framework in which inflammatory control and barrier-associated molecular restoration jointly contribute to the protective action of HJSS in CAG. This study provides a barrier-oriented mechanistic perspective for understanding the actions of multi-component herbal formulas in chronic gastric mucosal injury.

## Data Availability

The transcriptomic sequencing data used in this study have been deposited in the NCBI Sequence Read Archive database under BioProject accession number PRJNA1484638. The data can be accessed through the following link: https://www.ncbi.nlm.nih.gov/bioproject/1484638.
