# Supplementary material for: Huangjin Shuangshen decoction alleviates chronic atrophic gastritis by suppressing TNF/NF-κB signaling and promoting CFTR-associated gastric mucosal barrier repair
Source: Front Immunol. 2026 Jul 10;17:1848471. doi: 10.3389/fimmu.2026.1848471 (PMC13395671; doi:10.3389/fimmu.2026.1848471)
Supplement: Supplementary file 1 [file SupplementaryFile1.docx]

**
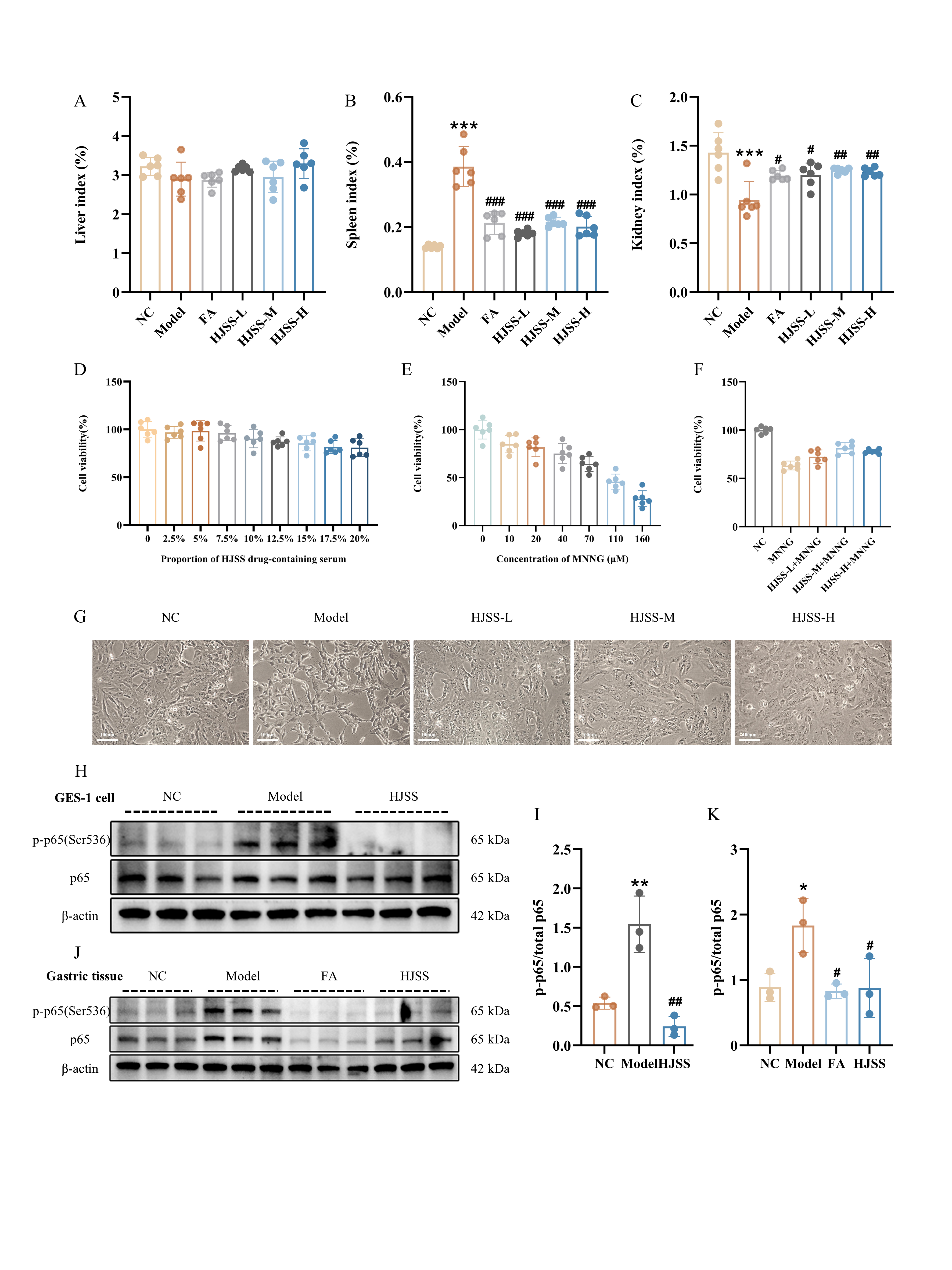
**

**Fig. S1. Supplementary assessment of organ indices, cell-model optimization, and p65 phosphorylation.**

(A–C) Organ indices of liver, spleen, and kidney. (D) Effects of different concentrations of HJSS-medicated serum on cell viability. (E) Effects of different concentrations of MNNG on cell viability. (F) Effects of HJSS-medicated serum on MNNG-induced loss of viability. (G) Representative cell morphology images. (H–I) Representative Western blot bands and quantitative analysis of p-p65 and total p65 expression in GES-1 cells. (J–K) Representative Western blot bands and quantitative analysis of p-p65 and total p65 expression in gastric tissues. Data are presented as mean ± SEM. For panels A–C, n = 6 mice per group; for panels D–F and H–I, n = 3 independent experiments; for panels J–K, n = 3 independent mouse samples per group. **P* < 0.05, ***P* < 0.01, ****P* < 0.001 vs. NC group; ^#^*P* < 0.05, ^##^*P* < 0.01, ^###^*P* < 0.001 vs. Model group.

**Table S1.** Information on antibodies.

| Antibody | Host species | Manufacturer | Cat**#** | Application |
| --- | --- | --- | --- | --- |
| ATP4B | Rabbit | Proteintech | 18692-1-AP | 1:500 for IHC |
| IL-6 | Rabbit | Abclonal | A0286 | 1:500 for IHC |
| IL-1β | Rabbit | Abclonal | A22257 | 1:500 for IHC |
| TNF-α | Rabbit | Abclonal | A28059 | 1:500 for IHC |
| Bax | Rabbit | Abclonal | A19684 | 1:2000 for IHC |
| Bcl2 | Rabbit | Abclonal | A19693 | 1:2000 for IHC |
| Caspase3 | Rabbit | Abclonal | A19664 | 1:500 for IHC |
| Ki67 | Rabbit | Abclonal | A20018 | 1:500 for IHC |
| CFTR | Mouse | Proteintech | 66928-1-Ig | 1:10000 for WB/1:500 for IHC/ 1:500 for IF |
| ZO-1 | Rabbit | Abclonal | A28491 | 1:2000 for WB/1:1000 for IHC/ 1:500 for IF |
| MUC5AC | Rabbit | Proteintech | 20725-1-AP | 1:1000 for IHC |
| Occludin | Rabbit | Abclonal | A24601 | 1:500 for IHC |
| Claudin-1 | Rabbit | Abclonal | A25902 | 1:300 for IHC |
| p65 | Rabbit | CST | 8242 | 1:1000 for WB/1:800 for IHC/ 1:400 for IF |
| p-p65(Ser536) | Rabbit | CST | 3033 | 1:10000 for WB |
| IκBα | Rabbit | Abclonal | A19714 | 1:5000 for WB |
| β-Tubulin | Rabbit | Abclonal | A12289 | 1:10000 for WB |
| Histone H3 | Rabbit | CST | 4499 | 1:1000 for WB |
| GAPDH | Mouse | Abclonal | AC033 | 1:10000 for WB |
